# Nonenzymatic Hydrogen Peroxide Detection Using Surface-Enhanced Raman Scattering of Gold–Silver Core–Shell-Assembled Silica Nanostructures

**DOI:** 10.3390/nano11102748

**Published:** 2021-10-17

**Authors:** Xuan-Hung Pham, Bomi Seong, Sungje Bock, Eunil Hahm, Kim-Hung Huynh, Yoon-Hee Kim, Wooyeon Kim, Jaehi Kim, Dong-Eun Kim, Bong-Hyun Jun

**Affiliations:** Department of Bioscience and Biotechnology, Konkuk University, Seoul 05029, Korea; phamricky@gmail.com (X.-H.P.); iambomi33@konkuk.ac.kr (B.S.); bsj4126@konkuk.ac.kr (S.B.); greenice@konkuk.ac.kr (E.H.); huynhkimhung82@gmail.com (K.-H.H.); hilite2201@naver.com (Y.-H.K.); buzinga5842@konkuk.ac.kr (W.K.); susia45@gmail.com (J.K.); kimde@konkuk.ac.kr (D.-E.K.)

**Keywords:** surface-enhanced Raman scattering, gold–silver core–shell, gold–silver core–shell-assembled silica nanostructure, hydrogen peroxide, peroxidase-mimicking catalytic activity, 3,3,5,5-tetramethylbenzidine

## Abstract

Hydrogen peroxide (H_2_O_2_) plays important roles in cellular signaling and in industry. Thus, the accurate detection of H_2_O_2_ is critical for its application. Unfortunately, the direct detection of H_2_O_2_ by surface-enhanced Raman spectroscopy (SERS) is not possible because of its low Raman cross section. Therefore, the detection of H_2_O_2_ via the presence of an intermediary such as 3,3,5,5-tetramethylbenzidine (TMB) has recently been developed. In this study, the peroxidase-mimicking activity of gold–silver core–shell-assembled silica nanostructures (SiO_2_@Au@Ag alloy NPs) in the presence of TMB was investigated using SERS for detecting H_2_O_2_. In the presence of H_2_O_2_, the SiO_2_@Au@Ag alloy catalyzed the conversion of TMB to oxidized TMB, which was absorbed onto the surface of the SiO_2_@Au@Ag alloy. The SERS characteristics of the alloy in the TMB–H_2_O_2_ mixture were investigated. The evaluation of the SERS band to determine the H_2_O_2_ level utilized the SERS intensity of oxidized TMB bands. Moreover, the optimal conditions for H_2_O_2_ detection using SiO_2_@Au@Ag alloy included incubating 20 µg/mL SiO_2_@Au@Ag alloy NPs with 0.8 mM TMB for 15 min and measuring the Raman signal at 400 µg/mL SiO_2_@Au@Ag alloy NPs.

## 1. Introduction

Since the discovery of the peroxidase-mimicking activity of iron (II, III) oxide (Fe_3_O_4_) on 3,3,5,5-tetramethylbenzidine (TMB), the development of enzyme-free H_2_O_2_ sensors with peroxidase-like activity has been accelerated [1]. Various nanostructured materials such as noble metal nanostructures [2,3,4,5,6,7,8], transition metal oxides [9,10,11], metal organic frameworks [12,13,14,15,16], and carbon-based nanostructures [17,18,19,20] have attracted great interest and have been successfully used to construct enzyme-free H_2_O_2_ sensors. Unlike native enzymes, nanomaterials possess many advantages, such as ease of preparation, favorable catalytic activity, low cost, and high stability. In particular, the properties of nanomaterials are retained when acting as a nanozyme [1,12,21]. However, the reactive intermediates adsorb to the surface of the nanomaterials and inhibit the catalytic reaction, which limits their applications [22]. In addition, the understanding of the structure/property relation of nanomaterials in H_2_O_2_ detection is still challenging, in vivo or in vitro [5,14]. Therefore, a sensitive and cost-effective material for detecting H_2_O_2_ must be developed. Noble metals such as Pt, Pd, Ag, and Au usually show excellent performance for H_2_O_2_ detection [23]. However, Au nanoparticles (NPs) show catalytic inefficiency in acidic media [12,24], and aggregation at high temperatures reduces their surface-to-volume ratio and limits their applications [3]. Therefore, the development of stable and high-efficiency nanomaterials as a nanozyme must be further investigated.

Recently, our group developed SiO_2_@Au@Ag alloy NPs using a combination of the seed growth method and a SiO_2_ template. The distance and uniformity of the Ag shell on SiO_2_ NPs was controlled by the Au seed, and the optical properties of the SiO_2_@Au@Ag alloy are tunable in the visible to near-infrared region [25,26,27]. Thus, SiO_2_@Au@Ag alloy exhibits a surface-enhanced Raman scattering (SERS) enhancement of 4.2 × 10^6^ with high reproducibility. The designing of an NP structure can be critical for SERS enhancement [28,29,30,31,32,33]. The SERS enhancement of the SiO_2_@Au@Ag alloy NPs is due to the enhancement of the cavity plasmon resonance and the variation of the refractive index of nanomaterials, by lead shifting the operation wavelength and enhancing the local electromagnetic fields of the hotspot region [29,30,33]. Because of their reliability and strength, SiO_2_@Au@Ag alloy-based substrates were developed for diagnosing cancers and detecting pesticides, thiram, and glucose [27,34,35,36,37,38,39]. However, their use in nanozymes and the detection of H_2_O_2_ using SERS requires further investigation.

The accurate detection of H_2_O_2_ is critical and has been gaining research attention as H_2_O_2_ plays important roles both in cellular signaling and in industry [40,41,42]. Various techniques, including chromatography [43], chemiluminescence [44], fluorescence [45,46], spectrophotometry [47], colorimetry [48], titrimetry [49], and SERS [2,50,51,52], have been used to detect H_2_O_2_. Although the electrochemical method is effective for the detection of H_2_O_2_, the lack of selectivity for interference such as that by oxidative species, because of the similarity of their potential to that of H_2_O_2_, limits its practical application [22]. Compared with the electrochemical method, SERS exhibits remarkable advantages, such as its indestructibility, fingerprinting, ultrasensitivity, and selectivity [53,54,55,56,57]. Therefore, various NPs, such as Ag and Au nanostructures as SERS substrates, have been fabricated for detecting H_2_O_2_ [2,50,51,52]. Unfortunately, the direct detection of H_2_O_2_ by SERS is not possible because of its low Raman cross section. Therefore, the detection of H_2_O_2_ via the presence of an intermediary such as TMB has recently been developed. More recently, Ag NPs have been utilized to increase the SERS signals in ELISA via the spontaneous aggregation of Ag NPs with positively charged oxidized TMB (oxTMB). This could be related to the concentration of some biomarkers such as human C-reactive protein or respiratory markers [58,59]. However, the underlying mechanism remains unclear.

**Scheme 1:**
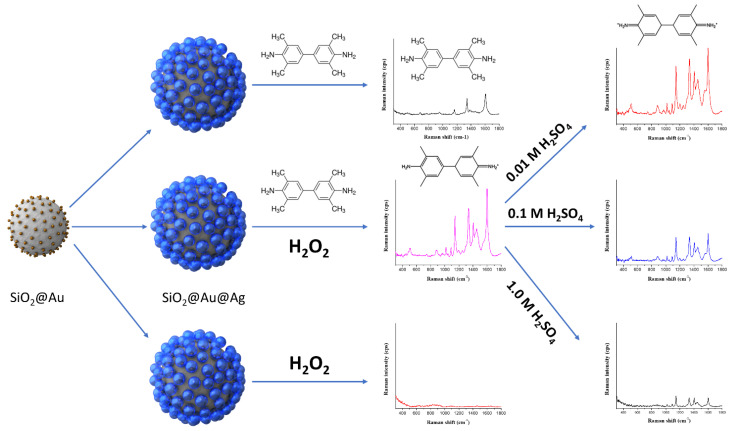
Illustration of the peroxidase-mimicking nanozyme activity of gold–silver core–shell-assembled silica alloy suspension (SiO_2_@Au@Ag alloy nanoparticles) in the presence of 3,3,5,5-tetramethylbenzidine and H_2_O_2_.

In this study, SiO_2_@Au@Ag alloy NPs were used as nanozymes and a SERS substrate to develop a method for detecting H_2_O_2_ via SERS, as outlined in Scheme 1. In the presence of H_2_O_2_, the SiO_2_@Au@Ag alloy NPs catalyze the conversion of TMB to oxTMB, adsorb onto the surface of the SiO_2_@Au@Ag alloy NPs, and demonstrate their SERS characteristics. As a result, the SERS band in the presence of H_2_O_2_ was evaluated by examining the SERS intensity of oxTMB bands on the surface of SiO_2_@Au@Ag alloy NPs. This result greatly expands the applicability of this technique for the detection of other biologically active targets based on the adsorption of TMB on SERS substrate.

## 2. Materials and Methods

### 2.1. Preparation of SiO_2_@Au@Ag Alloy NPs

SiO_2_@Au@Ag alloy NPs were prepared according to the steps outlined in a previous study [25]. Briefly, SiO_2_@Au seed was obtained by incubating 10 mL of Au NP suspension and 2 mL of aminated silica NPs overnight. The SiO_2_@Au@Ag alloy NPs were prepared by reducing 300 µM Ag_+_ to Ag on the surface of SiO_2_@Au in an aqueous medium using ascorbic acid in polyvinylpyrrolidone (PVP). The pellet was obtained by centrifuging the suspension for 15 min at 8500 rpm, washed thoroughly with EtOH, and re-dispersed in absolute EtOH to obtain a 200 µg/mL SiO_2_@Au@Ag alloy NP suspension.

### 2.2. Peroxidase-like Activity of SiO_2_@Au@Ag Alloy NPs in the TMB and H_2_O_2_ Mixture in Various Reaction Conditions

To verify the peroxidase-like activity of SiO_2_@Au@Ag alloy NPs, 10 mM TMB solutions were first prepared in EtOH. Next, 100 μL of TMB solution and 100 μL of SiO_2_@Au@Ag alloy NPs were added to 100 μL of phosphate-buffered saline (PBS) buffer (pH 7.0) containing freshly prepared H_2_O_2_ solution. The mixture was incubated for 15 min at 25 °C and centrifuged at 15,000 rpm for 15 min. The excess reagents were washed thoroughly with PBS containing 0.1% Tween 20 (PBST), and SiO_2_@Au@Ag@TMB NPs were then re-dispersed in PBST.

### 2.3. Optimal Peroxidase-like Activity of SiO_2_@Au@Ag in Various Reaction Conditions

#### 2.3.1. TMB Concentration

Solutions of various TMB concentration were prepared in EtOH. Next, 100 μL of TMB solution, 100 μL of 200 µg/mL SiO_2_@Au@Ag alloy NPs, and 100 μL of freshly prepared 2.0 M H_2_O_2_ solution were added to a pH 7.0 buffer (700 µL) to obtain final concentrations of TMB in the range of 0.1 to 1.0 mM. Each mixture was incubated for 15 min at 25 °C and centrifuged at 15,000 rpm for 15 min. The SiO_2_@Au@Ag@TMB NPs were then re-dispersed in PBST (100 µL) to obtain a SiO_2_@Au@Ag@TMB suspension.

#### 2.3.2. Reaction Time

The effect of reaction time on the SERS signal of SiO_2_@Au@Ag alloy NPs was investigated in the mixture containing 8.0 mM TMB solution (100 μL), SiO_2_@Au@Ag alloy NPs (0.2 mg/mL, 100 μL), and 2.0 M H_2_O_2_ solution (100 µL) in a pH 7.0 buffer (700 μL). The mixtures were reacted for 5 to 60 min. The prepared NPs were obtained by centrifuging at 15,000 rpm for 15 min, washed thoroughly, and redispersed in PBST.

#### 2.3.3. Amount of SiO_2_@Au@Ag Alloy NPs

To investigate the effect of SiO_2_@Au@Ag alloy NP amount on the SERS signal of SiO_2_@Au@Ag, 8.0 mM TMB solution (100 μL), 2.0 M H_2_O_2_ solution (100 µL) and SiO_2_@Au@Ag alloy NPs in the range of 10 to 50 µg were added in a pH 7.0 buffer (700 μL). The mixtures were reacted for 15 min at 25 °C, centrifuged for 15 min, washed thoroughly with PBST, and then re-dispersed in PBST (100 µL).

#### 2.3.4. pH Buffer

A 10 mM TMB solution (100 μL), SiO_2_@Au@Ag alloy NPs (0.2 mg/mL, 100 μL), and freshly prepared 2.0 M H_2_O_2_ solution (100 μL) were added to 700 mL of buffers at various pH values ranging from 3.0 to pH 9.0. Next, the mixtures were reacted for 15 min at 25 °C, centrifuged for 15 min, washed thoroughly with PBST, and then re-dispersed in PBST (100 µL).

#### 2.3.5. Concentration of SiO_2_@Au@Ag Alloy NPs for Raman Measurement

A 10 mM TMB solution (100 μL), SiO_2_@Au@Ag alloy NPs (0.2 mg/mL, 100 μL), and freshly prepared 2.0 M H_2_O_2_ solution (100 μL) were added to pH 6.0 buffer. Next, the mixtures were reacted for 15 min at 25 °C, centrifuged for 15 min, washed thoroughly with PBST, and then re-dispersed in of PBST. The volume of PBST was adjusted to obtain final concentrations of SiO_2_@Au@Ag alloy NPs in the range of 50 to 400 µg/mL for the Raman measurement.

#### 2.3.6. Detection of H_2_O_2_ using SiO_2_@Au@Ag Alloy NPs

A quantity of 20 µg SiO_2_@Au@Ag alloy NPs in PBST (100 µL) and 8.0 mM TMB in EtOH (100 µL) were added to pH 6.0 buffer (700 μL). PBS (100 µL) containing different concentrations of H_2_O_2_ (0.1 to 120 mM) was added to the above-mentioned mixture and allowed to react for 15 min at 25 °C. This mixture was centrifuged for 15 min at 15,000 rpm, washed thoroughly with PBST, and then re-dispersed in PBST to obtain a 400 µg/mL NP suspension for Raman measurement.

#### 2.3.7. Long-Term Stability of SiO_2_@Au@Ag Alloy NPs

To investigate the long-term stability of SiO_2_@Au@Ag alloy NPs, they were dispersed in EtOH at 200 µg/mL and stored at 4 °C for 60 days. The dispersion of SiO_2_@Au@Ag alloy NPs was shaken and diluted to 20 µg/mL. UV–vis spectroscopy was performed at wavelengths of 300 to 800 nm, and the absorbance of the SiO_2_@Au@Ag alloy NP suspension at 460 nm was recorded.

### 2.4. SERS Measurement

The SERS signals were measured at 10 mW for 5 s at randomly selected sites using a micro-Raman system with a 532 nm diode-pumped solid-state laser excitation source and an optical microscope (Olympus BX41, Tokyo, Japan) with a 10× objective lens (0.90 NA, Olympus, Tokyo, Japan). The laser beam spot was ~2.1 μm, and the SERS spectrum in the range of 300–1800 cm^−1^ was obtained.

## 3. Results and Discussion

SiO_2_@Au@Ag alloy NPs were prepared using a protocol reported by Pham et al. [27,34,35,36,37,38,39]. The transmission electron microscopy (TEM, JEOL, Akishima, Tokyo) images and UV–vis extinction spectra (Mecasys, Seoul, Korea) of the SiO_2_@Au@Ag alloy NPs are shown in Figure S1. The surface of SiO_2_@Au was effectively coated with the Ag shell. Various tiny Au NPs decorated the surfaces of the SiO_2_ NPs. The UV–vis extinction spectra of the SiO_2_@Au@Ag alloy NPs are consistent with the TEM images (Figure S1b). The as-prepared SiO_2_@Au@Ag alloy NP suspension shows the characteristic spectrum in Figure S1 with a broad band from 320 to 700 nm and a maximum peak at ~460 nm. This indicates that Ag shells formed on the SiO_2_@Au NP surfaces, created many hot-spot structures, and led a continuous spectrum of resonant multi-modes of the SiO_2_@Au@Ag alloy NP suspension [25,27]. These results are consistent with Mie’s theory, which states that an increase in particle size leads to a shift of the plasmon absorption band to longer wavelengths [60].

### 3.1. Peroxidase-Mimicking Nanozyme Activity of SiO_2_@Au@Ag Alloy NPs in the TMB–H_2_O_2_ Mixture

**Figure 1:**
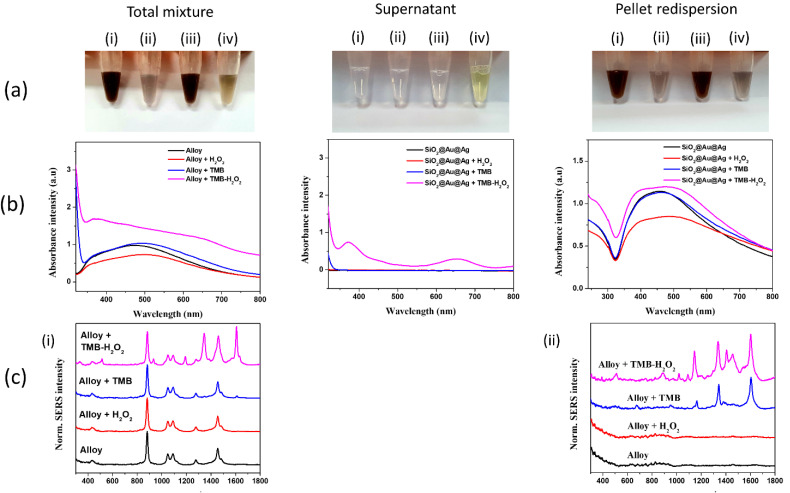
(**a**) Optical images and (**b**) ultraviolet–visible (UV–vis) extinction spectra of the peroxidase-mimicking nanozyme activity of the SiO_2_@Au@Ag alloy NP suspension (left column), pellet redispersion (right column), and supernatant (center column) in (i) the absence of TMB and H_2_O_2_ and in the presence of (ii) H_2_O_2_, (iii) TMB, and (iv) a mixture of TMB and H_2_O_2_. (**c**) Surface-enhanced Raman scattering (SERS) spectra of SiO_2_@Au@Ag alloy NPs in the TMB–H_2_O_2_ mixture in (i) EtOH and (ii) PBST at laser excitation of 532 nm. [H_2_O_2_] = 100 mM and [TMB] = 1 mM.

The peroxidase-like activity of the SiO_2_@Au@Ag alloy NPs was evaluated through the oxidation of TMB. The oxidation of TMB includes two steps: First, TMB is oxidized to TMB^+^ (oxTMB), and then the clear TMB solution changes to blue in color. However as TMB^+^ is quite unstable, it is oxidized to TMB^2+^ in acidic conditions and exhibits a yellow color [61]. In this study, we investigated the peroxidase-mimicking activity of SiO_2_@Au@Ag alloy NPs by mixing 20 µg SiO_2_@Au@Ag alloy NPs (i) in 100 µL of H_2_O_2_ (ii), TMB (iii), and TMB–H_2_O_2_ mixture (iv) as shown in Figure 1a. The reaction solutions were incubated for 15 min at 25 °C, and the results are shown in Figure 1a. The color of the SiO_2_@Au@Ag alloy NP suspension in TMB solution (left column, (iii)) was dark brown and similar to that of SiO_2_@Au@Ag (left column, (i)), whereas the SiO_2_@Au@Ag alloy NP suspension in H_2_O_2_ solution (left column, ii) turned grey in color due to its oxidation by H_2_O_2_ solution. In the presence of TMB and H_2_O_2_, the color of the SiO_2_@Au@Ag alloy NP suspension was light brown (left column, iv). After centrifugation, the supernatant in TMB and H_2_O_2_ solution in the presence of SiO_2_@Au@Ag alloy NPs was transparent and colorless (center column, (ii) and (iii)), indicating that peroxidase-mimicking activity did not occur in the absence of either H_2_O_2_ or TMB. By contrast, the supernatant in the TMB–H_2_O_2_ mixture and SiO_2_@Au@Ag alloy NPs (center column, (iv)) changed in color from transparent to yellow as shown in Figure 1a(i–iv). The colors of the pellets of SiO_2_@Au@Ag alloy NPs after centrifugation and redispersion in PBST are shown in the right-hand column of Figure 1a. Once again, the color of SiO_2_@Au@Ag alloy NP suspension (i) was the same as that of SiO_2_@Au@Ag alloy NPs with TMB solution (iii), whereas the SiO_2_@Au@Ag alloy NP suspensions in H_2_O_2_ (ii) and a mixture of TMB and H_2_O_2_ (iv) became light grey and dark grey, respectively. These results imply that SiO_2_@Au@Ag alloy NPs possess peroxidase-mimicking activity in the presence of H_2_O_2_ during the conversion of TMB to oxTMB.

The UV–vis extinction spectra of the SiO_2_@Au@Ag alloy NP suspension before and after centrifugation are shown in Figure 1b. The SiO_2_@Au@Ag alloy NP suspension without TMB and H_2_O_2_ showed a broad band from 320 nm to 700 nm with a maximum peak at ~460 nm. The presence of either TMB or H_2_O_2_ led the UV–vis extinction spectra of the SiO_2_@Au@Ag alloy NPs to be slightly red-shifted from ~460 nm to ~500 nm (left column), whereas the suspension of SiO_2_@Au@Ag alloy NPs in TMB–H_2_O_2_ mixture showed a broad and strong peak in the range of 350 nm to 800 nm with clear and multiple peaks at 370 nm and 650 nm [62]. This indicated that TMB was converted to oxTMB in the presence of H_2_O_2_; this reaction was catalyzed by SiO_2_@Au@Ag alloy NPs, as shown in Figure S2. Moreover, the supernatant of the SiO_2_@Au@Ag alloy NPs in TMB–H_2_O_2_ mixture also confirmed the presence of oxTMB when the SiO_2_@Au@Ag alloy NPs were removed from the suspension because of the excess oxTMB in the supernatant, which did not adsorb onto the surface of SiO_2_@Au@Ag alloy NPs (center column). Therefore, SiO_2_@Au@Ag alloy NPs possessed an intrinsic peroxidase-mimicking activity that catalyzed the conversion of TMB to oxTMB, as expected. In addition, the zeta potential values of the SiO_2_@Au@Ag alloy NPs in TMB, H_2_O_2_, and a mixture of TMB and H_2_O_2_ were also studied, as shown in Figure S3a. SiO_2_@Au@Ag alloy NPs showed a zeta potential of −24.5 ± 0.6 mV due to the rich electron cloud of the Ag layer. The presence of H_2_O_2_ or TMB in the suspension of SiO_2_@Au@Ag alloy NPs converted the surface charge to −14.6 ± 0.4 mV or −2.3 ± 0.6 mV, respectively. Thus, the zeta potential of SiO_2_@Au@Ag alloy NPs increased from −24.5 ± 0.6 mV to −14.6 ± 0.1 mV after adding the TMB–H_2_O_2_ mixture into the reaction mixture.

In EtOH solution, the Raman spectra of the SiO_2_@Au@Ag alloy NP suspension in TMB and H_2_O_2_ are shown in Figure 1c(i). In the SiO_2_@Au@Ag alloy NP suspension, SERS bands were obtained at 431, 883, 1049, 1093, 1277, and 1455 cm^−1^, which were assigned to the EtOH solution [27]. New SERS bands of the TMB–H_2_O_2_ mixture in the presence of SiO_2_@Au@Ag alloy NPs were also observed at 510, 1191, 1341, 1463, and 1608 cm^−1^. The bands of the TMB–H_2_O_2_ mixture at 1341, 1463, and 1608 cm^−1^ were remarkably increased compared to those of the TMB solution. However, the overlap in some EtOH and oxTMB bands and the high background signal of EtOH can hinder analysis and give false results. Therefore, SiO_2_@Au@Ag alloy NPs must be centrifuged and re-dispersed in PBST, and the Raman spectra must be measured in the PBST solution. Indeed, the SERS bands of EtOH disappeared, and the SERS bands of TMB and oxTMB are clearly observed in Figure 1c(ii). The TMB bands showed typical SERS bands at 1191, 1341, and 1608 cm^−1^, which correspond to the CN stretching vibration, CH stretching vibration, and CC stretching vibration, respectively [59,62]. The SERS bands of SiO_2_@Au@Ag alloy NPs in the TMB–H_2_O_2_ mixture were observed at 1191, 1341, 1468, 1563, and 1608 cm^−1^. In contrast with the results of a previous study [58,59], oxTMB in our study did not induce the aggregation of Ag NPs but was adsorbed on the surface of SiO_2_@Au@Ag alloy NPs and showed the characteristic bands of oxTMB as mentioned above. Therefore, these Raman peaks were all used for the measurement of the SiO_2_@Au@Ag alloy NPs + TMB–H_2_O_2_ system.

**Figure 2:**
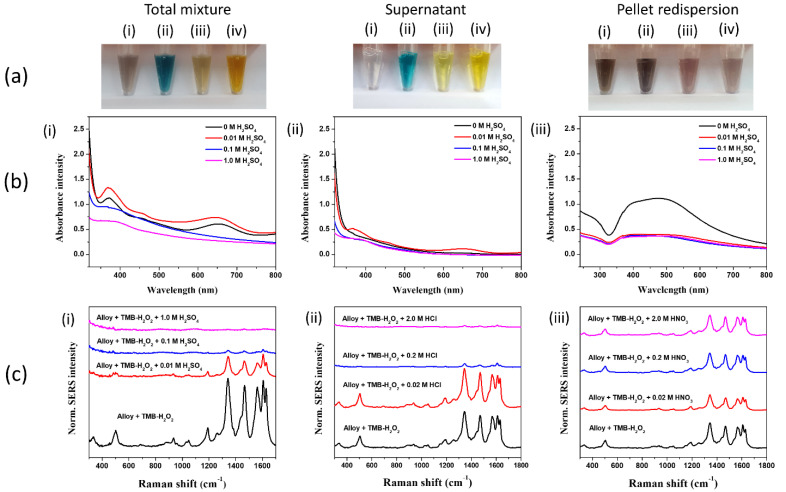
(**a**) Optical images of SiO_2_@Au@Ag alloy suspension in the TMB–H_2_O_2_ mixture after adding (i) 0 M, (ii) 0.01 M, 0.1 M, and 1.0 M H_2_SO_4_. (**b**) UV–vis extinction spectra of (i) SiO_2_@Au@Ag alloy total suspension, (ii) supernatant, and (iii) pellet redispersion in the mixture of TMB and H_2_O_2_ at various concentrations of H_2_SO_4_. (**c**) SERS spectra of SiO_2_@Au@Ag alloy suspension in the mixture of TMB and H_2_O_2_ after the addition of (i) H_2_SO_4_, (ii) HCl, and (iii) HNO_3_. [H_2_O_2_] = 100 mM and [TMB] = 1 mM.

According to the literature [61,63], peroxidase activity was stopped by adding H_2_SO_4_ to convert oxTMB to TMB^2+^, which is stable under acidic conditions and possesses an absorbance at 455 nm (Figure S2). The colors of the mixtures of SiO_2_@Au@Ag alloy NPs and TMB–H_2_O_2_ after adding H_2_SO_4_ are shown in Figure 2a (left column). The suspension changed from brown to blue-green at low H^+^ concentration (0.01 M H_2_SO_4_), indicating that oxTMB can partly convert to TMB^2+^, giving a mix of blue (oxTMB) and yellow (TMB^2+^ in acidic conditions). By contrast, the colors of the mixtures of SiO_2_@Au@Ag alloy NPs and TMB–H_2_O_2_ changed from brown to light yellow and yellow, respectively, when 0.1 M and 1.0 M H_2_SO_4_ were added to the reaction suspension (Figure 2a). This indicates that oxTMB can be completely converted to TMB^2+^. The results were confirmed by the color of supernatant after centrifugation in Figure 2a (center column).

The UV–vis extinction spectra of the SiO_2_@Au@Ag alloy NPs suspensions in TMB–H_2_O_2_ mixture + H_2_SO_4_ are consistent with the optical images in Figure 2b. At low H^+^ concentrations, the peaks of oxTMB at 370 and 650 nm are clearly observed in Figure 2b(i,ii). These two peaks disappeared after the addition of 0.1 M and 1.0 M H_2_SO_4_, indicating the conversion of oxTMB to TMB^2+^. However, the absorbance intensity of the SiO_2_@Au@Ag alloy NP pellet collected after centrifugation and redispersion in PBST dramatically decreased with the addition of H_2_SO_4_ (Figure 2b(iii)).

Furthermore, the SERS signal of SiO_2_@Au@Ag alloy NP suspension in TMB–H_2_O_2_ + H_2_SO_4_ sharply decreased with an increase in H_2_SO_4_ concentration from 0.01 M to 1.0 M (Figure 2c(i)). The decrease in the SERS signal of oxTMB following the addition of H_2_SO_4_ was caused by the desorption of oxTMB from the surface of SiO_2_@Au@Ag alloy NPs and/or low enhancement of the Ag substrate due to the formation of Ag_2_SO_4_ on the surface of SiO_2_@Au@Ag alloy NPs. To examine the charge of SiO_2_@Au@Ag alloy NPs in TMB–H_2_O_2_ + H_2_SO_4_, the zeta potential of SiO_2_@Au@Ag alloy NPs in the TMB–H_2_O_2_ mixture before and after adding H_2_SO_4_ was measured, as shown in Figure S3b. The zeta potential of SiO_2_@Au@Ag alloy NPs increased from −14.6 ± 0.1 mV to −4.4 ± 0.4 mV after the addition of 1.0 M H_2_SO_4_ to the TMB–H_2_O_2_ mixture. This result indicates that TMB^2+^ could still be immobilized on the surface of SiO_2_@Au@Ag alloy NPs. Therefore, we changed the acidic agent from H_2_SO_4_ to HCl and HNO_3_, while retaining the H^+^ concentration at 2.0 M in the reaction. Similar to that with H_2_SO_4_, the SERS signal of oxTMB in the SiO_2_@Au@Ag alloy NP suspension also decreased slightly with an increase in HCl concentration, possibly because of the formation of AgCl on the surface of SiO_2_@Au@Ag alloy NPs (Figure 2c(ii)). By contrast, the SERS signal of oxTMB in SiO_2_@Au@Ag alloy NP suspension remained almost the same when 0.2 M and 2.0 M HNO_3_ were added. However, the SERS signal of oxTMB slightly decreased when 0.02 M HNO_3_ was added to the reaction. This was because the highly water-soluble AgNO_3_ was dissolved in the aqueous solution and was not adsorbed on the surface of the SiO_2_@Au@Ag alloy NPs. Thus, the decrease in the SERS signal of SiO_2_@Au@Ag alloy NPs in TMB–H_2_O_2_ mixture after adding H_2_SO_4_ was caused by the formation of Ag_2_SO_4_, and it decreased the electromagnetic enhancement of the Ag layer on the surface of the SiO_2_@Au@Ag alloy NPs_._ Therefore, we decided not to use acidic conditions to terminate the peroxidase reaction of SiO_2_@Au@Ag alloy NPs.

### 3.2. Effect of Ag^+^ Concentration on the Detection of H_2_O_2_ by SiO_2_@Au@Ag Alloy NPs

**Figure 3:**
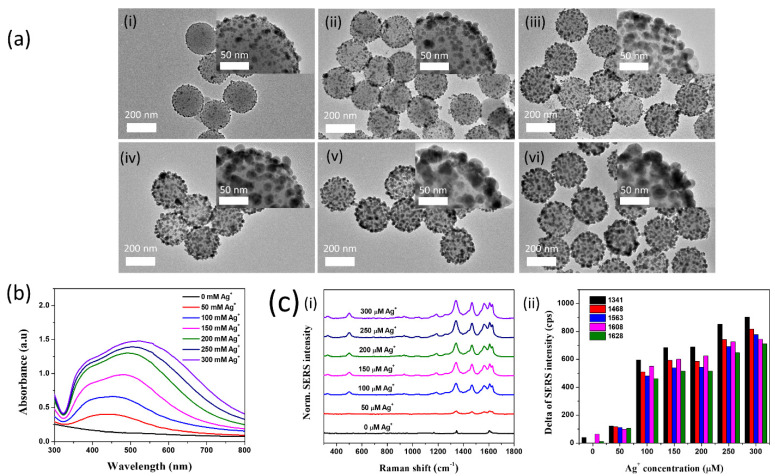
(**a**) Transmission electron microscopy images and (**b**) UV–vis extinction spectra of SiO_2_@Au@Ag alloy NPs synthesized at various concentration of Ag^+^: (i) 50, (ii) 100, (iii) 150, (iv) 200, (v) 250, and (vi) 300 µM Ag^+^. (**c**) (i) SERS curves and (ii) SERS signal plot of SiO_2_@Au@Ag alloy NPs in TMB–H_2_O_2_ mixture at various concentration of Ag^+^.

According to our previous report, the SERS enhancement of the SiO_2_@Au@Ag alloy NPs depends on the gaps between Ag NPs on the surface of SiO_2_@Au@Ag alloy NPs, as indicated by adjusting the concentration of AgNO_3_ in the solution from 50 to 300 µM [27,38,39]. In Figure 3a, the size of the Au@Ag increased with the concentration of Ag^+^ used.

The UV–vis extinction spectra of the SiO_2_@Au@Ag alloy NPs shown in Figure 3b are in agreement with the results of TEM images. The maximum UV–vis extinction peak was red-shifted from 450 to 530 nm and broadened from 300 to 800 nm with an increase of Ag^+^ because of the generation of hot-spot structures between two adjacent Au@Ag NPs on the SiO_2_@Au@Ag alloy NPs [22,50,64].

The peroxidase-mimicking activities of SiO_2_@Au@Ag alloy NPs prepared at various Ag^+^ concentrations toward the TMB–H_2_O_2_ mixture are shown in Figure 3c. The SERS signal intensities at 1314, 1468, 1563, 1608, and 1628 cm^−1^ were proportional to the AgNO_3_ concentration and highest at 300 µM due to the narrow gaps between Ag NPs on the SiO_2_@Au@Ag alloy NP surfaces that created “hot-spots” and strongly enhanced the electromagnetic field surrounding the SiO_2_@Au@Ag alloy NPs [33,65]. At higher Ag^+^ concentration, the SiO_2_@Au@Ag alloy NPs were aggregated in the TMB–H_2_O_2_ mixture. Therefore, 300 µM AgNO_3_ was the optimal concentration for synthesizing SiO_2_@Au@Ag alloy NPs with the highest peroxidase-mimicking activity.

### 3.3. Optimization of SiO_2_@Au@Ag Alloy NPs for Detecting H_2_O_2_

**Figure 4:**
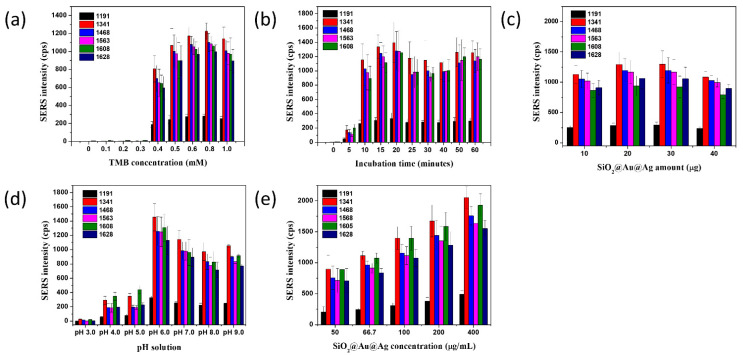
Optimization of H_2_O_2_ detection using the SiO_2_@Au@Ag alloy NPs in the presence of TMB; SERS signal plot at 1191, 1341, 1468, 1563, 1608, and 1628 cm^−1^ of H_2_O_2_ at various (**a**) concentrations of TMB ranging from 0.1 to 1.0 mM, (**b**) incubation times, (**c**) amounts of SiO_2_@Au@Ag alloy NPs, (**d**) solution pH, and (**e**) dilutions of SiO_2_@Au@Ag alloy NPs for Raman measurement in PBST.

In the literature, the catalytic activity of nanozymes is also affected by reaction conditions [1,66,67,68,69,70]. The effects of the reaction conditions, including TMB concentration, reaction time, number of SiO_2_@Au@Ag alloy NPs, and pH of the buffer, on the peroxidase-mimicking activity of SiO_2_@Au@Ag alloy NPs were considered in this study. As mentioned above, the SERS bands of oxTMB at 1191, 1341, 1468, 1563, 1608, and 1628 cm^−1^ were investigated in the TMB–H_2_O_2_ mixture. The effects of these conditions on the SERS signal of the SiO_2_@Au@Ag alloy NPs in the TMB–H_2_O_2_ mixture were examined and optimized, as shown in Figure 4.

To establish the effect of TMB concentration on the SERS signal of the SiO_2_@Au@Ag alloy NPs in the TMB–H_2_O_2_ mixture, the concentrations of TMB were investigated in the range of 0 to 1.0 mM (Figure 4a). The SERS signal of SiO_2_@Au@Ag alloy NPs was almost insignificant at TMB concentrations lower than 0.4 mM. It increased remarkably when the TMB concentration was higher than 0.4 mM, achieved the highest value at 0.8 mM, but then decreased at higher TMB concentrations because of the poor solubility of TMB in aqueous solution (Figure 4a) [63].

The reaction time or incubation time for detecting H_2_O_2_ using SiO_2_@Au@Ag alloy NPs is also shown in Figure 4b. The SERS signals of oxTMB at 1191, 1341, 1468, 1563, 1608, and 1628 cm^−1^ increased with reaction time and were saturated at 20 min. A gradual decrease in the SERS signal of SiO_2_@Au@Ag alloy NPs occurred after 20 min, owing to the instability of oxTMB [61].

In addition, the effect of the amount of SiO_2_@Au@Ag alloy NPs was observed in the range of 10–40 µg, as shown in Figure 4c. The SERS signal is dependent on the Raman reporter density on the nanomaterial surface [26]. Therefore, when a large amount of SiO_2_@Au@Ag alloy NPs was added, less oxTMB was available on the surface of the SiO_2_@Au@Ag alloy NPs. The SERS signals at all SERS bands of SiO_2_@Au@Ag alloy NPs in the TMB–H_2_O_2_ mixture decreased when 40 µg of SiO_2_@Au@Ag alloy NPs was used, indicating that the density of oxTMB gradually decreased with an increase in the number of substrates. The SERS signals at all SERS bands of SiO_2_@Au@Ag alloy NPs increased with a decrease in alloy quantity, as shown in Figure 4c. However, a small amount of SiO_2_@Au@Ag alloy NPs reduces the peroxidase-mimicking catalytic efficiency of the SiO_2_@Au@Ag alloy NPs to convert TMB to oxTMB and lower the SERS signal of oxTMB at 10 µg (Figure 4c). Therefore, to ensure that the SiO_2_@Au@Ag alloy NPs can convert sufficient amounts of TMB to oxTMB in the presence of H_2_O_2_, we decided to use 20 µg of SiO_2_@Au@Ag alloy NPs for further study.

The effect of the pH on the SERS signal of SiO_2_@Au@Ag alloy NPs in the TMB–H_2_O_2_ mixture is shown in Figure S4 and Figure 4d. Similar to that of Au, the catalytic activity of SiO_2_@Au@Ag alloy NPs on H_2_O_2_ was also pH-dependent. The SERS signal of SiO_2_@Au@Ag alloy NPs in the TMB–H_2_O_2_ mixture showed the highest peroxidase-mimicking activity at pH 6.0. At pH ≤ 4, the SERS signal of the TMB–H_2_O_2_ mixture was too weak in all SERS bands. The SERS signal increased in the pH range of 5–6 and then decreased in the pH range of 7–9. In previous reports, the catalytic reaction of TMB–H_2_O_2_ was much faster in a weakly acidic solution than in neutral or basic solutions. However, in our study, the SiO_2_@Au@Ag alloy NPs lost 95% of their maximum activity at pH 3.0 and retained ~70% of their maximum activity in the pH range of 7.0–9.0. This is because SiO_2_@Au@Ag alloy NPs catalyze the generation of ·OH from the decomposition of H_2_O_2_ with the dissolution of Ag to release Ag^+^ under strongly acidic conditions, as shown in the following equation:

H_2_O_2_ + 2Ag + 2H^+^ → 2H_2_O + 2Ag^+^

The enhancement of the H_2_O_2_-reducing ability at high pH led to an increase in the SERS signal of oxTMB. However, under basic conditions, the Ag layer on the surface of SiO_2_@Au@Ag alloy NPs can be converted to Ag(OH) or Ag_2_O, which lowers the signal enhancement of oxTMB on SiO_2_@Au@Ag alloy NPs.

For Raman measurement in the liquid phase using a capillary tube, the concentration of the SERS substrate during Raman measurement strongly affects the SERS signal [26,36,38]. Figure 4d shows the effect of the concentration of SiO_2_@Au@Ag alloy NPs after incubation in the TMB–H_2_O_2_ mixture. In the absence of H_2_O_2_, the SERS signal of the SiO_2_@Au@Ag alloy NP suspension decreased slightly with increasing concentration of SiO_2_@Au@Ag alloy NPs for Raman measurements. Moreover, the SERS signals of SiO_2_@Au@Ag alloy NPs in the TMB–H_2_O_2_ mixture increased slightly when the concentration of SiO_2_@Au@Ag alloy NPs decreased sharply. The SERS signal achieved the highest value at 400 µg/mL. Thus, the optimal condition for H_2_O_2_ detection by SiO_2_@Au@Ag alloy NPs in TMB–H_2_O_2_ mixture was achieved at 8 mM TMB for 15 min reaction with 20 µg SiO_2_@Au@Ag alloy NPs, and Raman measurement was performed at 400 µg/mL SiO_2_@Au@Ag alloy NPs.

### 3.4. Calibration Curve for Detecting H_2_O_2_

**Figure 5:**
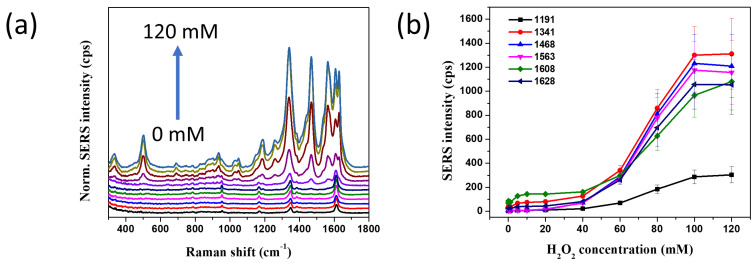
(**a**) SERS signal curves and (**b**) SERS plot of SiO_2_@Au@Ag alloy NPs in TMB–H_2_O_2_ mixture at various concentrations of hydrogen peroxide from 0 to 120 mM under optimized conditions, which included incubation with 20 µg SiO_2_@Au@Ag alloy NPs and 0.8 mM TMB for 15 min and Raman measurement at 400 µg/mL SiO_2_@Au@Ag alloy NPs.

At optimal conditions, the SERS spectra of SiO_2_@Au@Ag alloy NPs in the presence of TMB were recorded at various concentrations of H_2_O_2_. Variation in the SERS signal of SiO_2_@Au@Ag alloy NPs in the TMB–H_2_O_2_ mixture was obtained in concentrations of H_2_O_2_ from 0.1 to 120 mM (Figure 5a). The SERS signals at 1191, 1341, 1468, 1563, 1608, and 1628 cm^−1^ gradually increased when the concentration of H_2_O_2_ was lower than 20 mM (Figure 5b). However, they increased remarkably in the range of 40 to 100 mM H_2_O_2_. This implies that H_2_O_2_ efficiently converted TMB to oxTMB and that oxTMB was immobilized on the surface of the SiO_2_@Au@Ag alloy NPs. The SERS peak reached saturation with an increase in H_2_O_2_ concentration. This result indicates a complete coverage of oxTMB on the SiO_2_@Au@Ag alloy NP surfaces. However, the SERS band of oxTMB showed a shift at high H_2_O_2_ concentration due to the formation of a dimer, a trimer, or the twist of oxTMB on the surface of the SiO_2_@Au@Ag alloy NPs [71]. Therefore, we concluded that the SERS signal of SiO_2_@Au@Ag alloy NPs in H_2_O_2_ solution in the presence of TMB was the result of the catalytic activity involved in the conversion of TMB to oxTMB and the adsorption of oxTMB on the SiO_2_@Au@Ag alloy NP surfaces.

The calibration of H_2_O_2_ detection was performed via linear curve fitting in the experimental data points ranging from 40 to 100 mM (Figure S5). A significant linear relationship of y = 20.04x + 802.17 was found between the SERS signals and H_2_O_2_ concentration, where x is the H_2_O_2_ concentration and y is the SERS signal at 1468 cm^−1^ (R^2^ = 0.98). The theoretical limit of detection was 33.3 mM, as estimated using the 3s blank criterion.

The effect of long-term storage of SiO_2_@Au@Ag alloy NPs is shown in Figure S6. SiO_2_@Au@Ag alloy NPs (200 µg/mL) were stored at 4 °C for 60 days. The UV–vis spectra of the SiO_2_@Au@Ag alloy NPs were measured at the desired time, and the absorbance at 450 nm was monitored. As shown in Figure S7, the SERS signal was stable for 60 days.

## 4. Conclusions

We developed a SERS-based H_2_O_2_ detection method using SiO_2_@Au@Ag alloy NPs in the presence of TMB. In this work, we demonstrated that TMB was converted to oxTMB by the SiO_2_@Au@Ag alloy NPs in the presence of H_2_O_2_ and that oxTMB was absorbed on the surface of SiO_2_@Au@Ag alloy NPs. We also provide a calibration curve to evaluate H_2_O_2_ species in the range of 40 to 100 mM with a limit of detection of 33.3 mM. Moreover, the optimal conditions for H_2_O_2_ detection using SiO_2_@Au@Ag alloy NPs include incubating 20 µg/mL SiO_2_@Au@Ag alloy NPs with 0.8 mM TMB for 15 min and measuring the Raman signal at 400 µg/mL of SiO_2_@Au@Ag alloy NPs. Even though the limit of detection of our structure is not low, it acted as both a nanozyme and a SERS substrate for the adsorption of TMB. This result greatly expands its applicability for the detection of other biologically active targets.

## Data Availability

Data is contained within the article or supplementary material.

## Supplementary Materials

The following are available online at https://www.mdpi.com/article/10.3390/nano11102748/s1, Figure S1. (a) Transmission electron microscopy images and (b) ultraviolet–visible extinction spectra of (i) SiO_2_@Au (1 mg/mL) and (ii) SiO_2_@Au@Ag alloy nanoparticles (NPs) (20 µg/mL) synthesized using 2 mg SiO_2_@NH_2_ and 300 mM Ag^+^. Figure S2. Schematic illustration of the catalytic mechanism of SiO_2_@Au@Ag alloy NPs in the TMB–H_2_O_2_ mixture. TMB is oxidized to oxTMB by SiO_2_@Au@Ag alloy NPs that act as peroxidase in the presence of H_2_O_2_. Next, oxTMB is converted to TMB^2+^ in the acidic condition. Figure S3. (a) Zeta potential of SiO_2_@Au@Ag alloy NPs alone and SiO_2_@Au@Ag alloy NPs in the presence of H_2_O_2_, TMB, and a mixture of TMB and H_2_O_2_. (b) Zeta potential of SiO_2_@Au@Ag alloy NPs in a mixture of TMB and H_2_O_2_ before and after the addition of H_2_SO_4_. Figure S4. Surface-enhanced Raman spectroscopy (SERS) signals of SiO_2_@Au@Ag alloy NPs in various pH solutions, with pH ranging from 3.0 to 9.0 in the TMB–H_2_O_2_ mixture. Figure S5. Calibration curves of SERS signal at (a) 1191, (b) 1341, (c) 1468, (d) 1563, (e) 1608, and (f) 1628 cm^−1^ of SiO_2_@Au@Ag alloy NPs in the TMB–H_2_O_2_ mixture with the concentrations of H_2_O_2_ ranging from 40 to 100 mM. Figure S6. Long-term storage of 200 µg/mL SiO_2_@Au@Ag alloy NPs at 4 °C in ethanol solution.
