# Supplementary material for: Nonenzymatic Hydrogen Peroxide Detection Using Surface-Enhanced Raman Scattering of Gold–Silver Core–Shell-Assembled Silica Nanostructures
Source: Nanomaterials (Basel). 2021 Oct 17;11(10):2748. doi: 10.3390/nano11102748 (PMC8540490; doi:10.3390/nano11102748)
Supplement: Supplementary file 1 [file nanomaterials-11-02748-s001.zip › nanomaterials-1389419-supplementary.pdf]

Article

Nonenzymatic Hydrogen Peroxide Detection Using Surface-Enhanced Raman Scattering of Gold–Silver Core–Shell-Assembled Silica Nanostructures

Xuan-Hung Pham, Bomi Seong, Sungje Bock, Eunil Hahm, Kim-Hung Huynh, Yoon-Hee Kim, Wooyeon Kim, Jaehi Kim, Dong-Eun Kim and Bong-Hyun Jun *

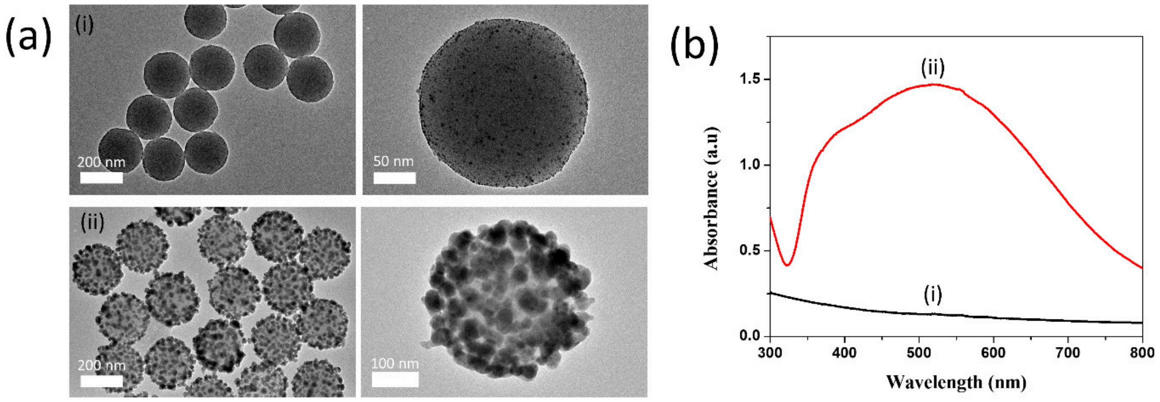

Figure S1. (**a**) Transmission electron microscopy images and (**b**) ultraviolet–visible extinction Scheme 2. Au (1 mg/mL) and (ii) SiO_2@Au@Ag alloy nanoparticles (NPs) (20 μg/mL) synthesized using 2 mg SiO_2@NH_2 and 300 μM Ag^+.

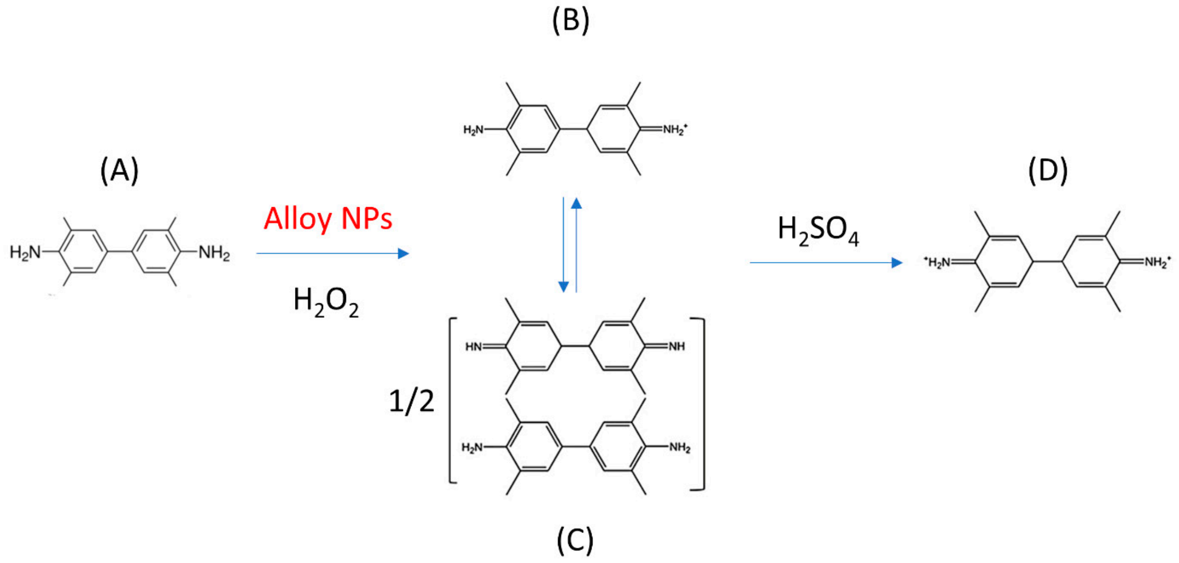

A: TMB
B: TMB^{+}
C: Charge transfer complex of TMB
D: TMB^{2+}
Alloy NPs: SiO_2@Au@Ag

Figure S2. Schematic illustration of the catalytic mechanism of SiO_2@Au@Ag alloy NPs in the TMB–H_2O_2 mixture. TMB is oxidized to oxTMB by SiO_2@Au@Ag alloy NPs that act as peroxidase in the presence of H_2O_2. Next, oxTMB is converted to TMB^{2+} in the acidic condition.

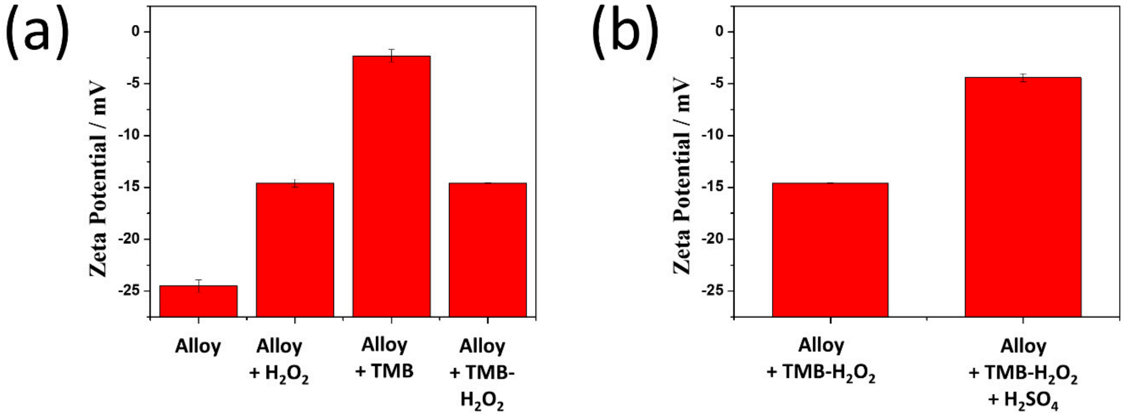

Figure S3. (**a**) Zeta potential of SiO_2@Au@Ag alloy NPs alone and SiO_2@Au@Ag alloy NPs in the presence of H_2O_2, TMB, and a mixture of TMB and H_2O_2. (**b**) Zeta potential of SiO_2@Au@Ag alloy NPs in a mixture of TMB and H_2O_2 before and after the addition of H_2SO_4.

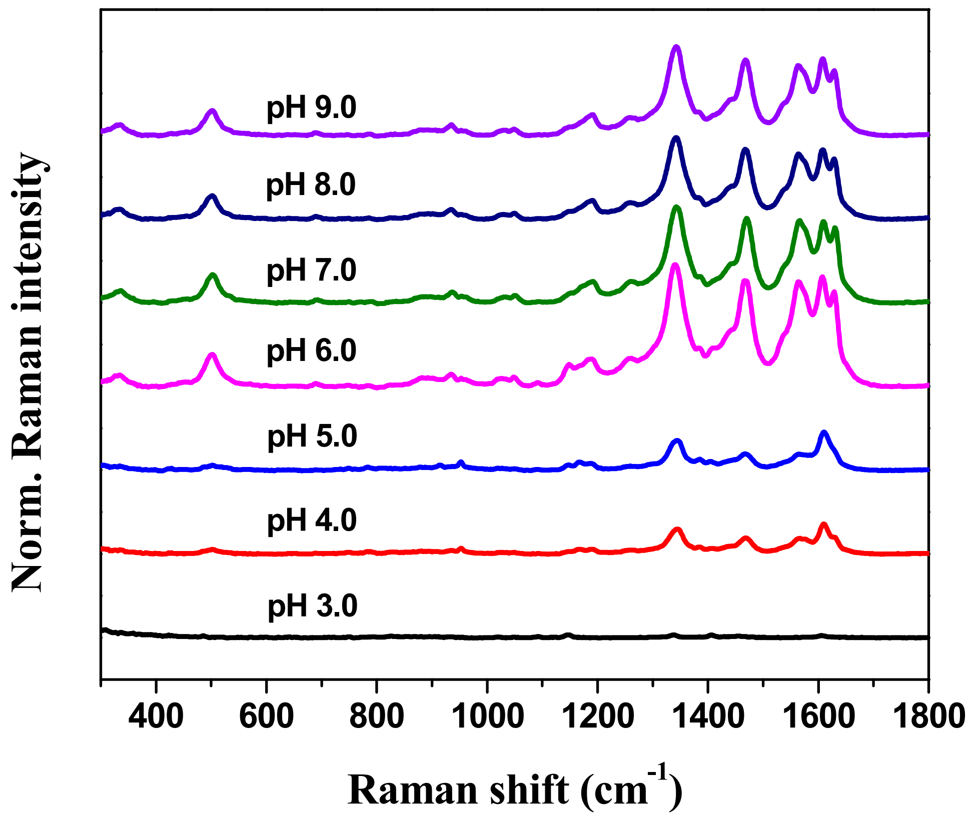

Figure S4. Surface-enhanced Raman spectroscopy signals of SiO_2@Au@Ag alloy NPs in various pH solutions, with pH ranging from 3.0 to 9.0 in the TMB–H_2O_2 mixture.

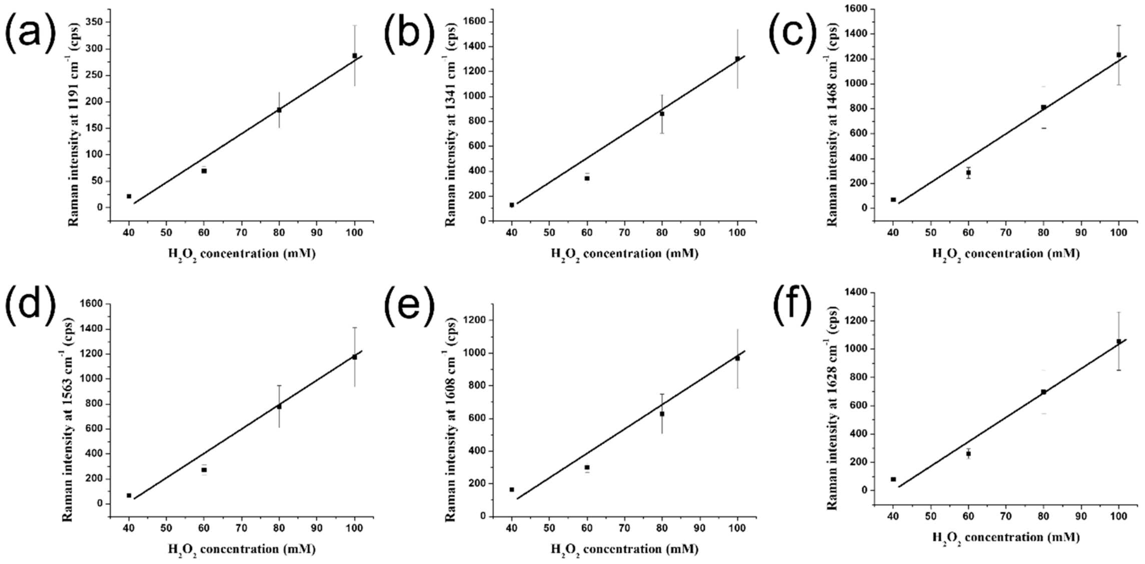

Figure S5. Calibration curves of SERS signal at (a) 1191, (b) 1341, (c)1468, (d) 1563, (e) 1608, and (f) 1628 cm^{-1} of SiO_2@Au@Ag alloy NPs in the TMB–H_2O_2 mixture with the concentrations of H_2O_2 ranging from 40 to 100 mM.

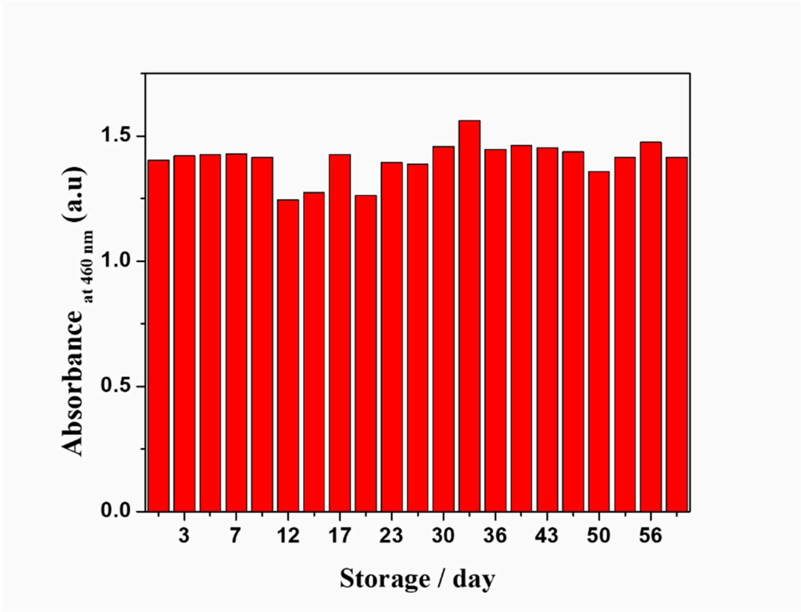

Figure S6. Long-term storage of 200 μg/mL SiO_2@Au@Ag alloy NPs at 4 °C in ethanol solution.
